# COVID-19 Racism, Depressive Symptoms, Drinking to Cope Motives, and Alcohol Use Severity Among Asian American Emerging Adults

**DOI:** 10.1177/21676968221117421

**Published:** 2022-12

**Authors:** Brian TaeHyuk Keum, Andrew Young Choi

**Affiliations:** 1 Department of Social Welfare, 1068 University of California, Los Angeles, CA, USA; 2 Counseling and Student Development Center, University of Hawaiʻi at Mānoa, HI, USA

**Keywords:** Alcohol use severity, Asian American emerging adults, COVID-19 racism, depressive symptoms, drinking to cope motives

## Abstract

The COVID-19 pandemic has incited widespread anti-Asian racism, which is linked to numerous behavioral health consequences including depressive symptoms. As racism-induced depressive symptoms are linked to coping-related alcohol use and because alcohol-related problems represent a significant public health concern in this population, we investigated whether COVID-19 racism predicted alcohol use severity through depressive symptoms and drinking to cope motives among Asian American emerging adults (*N* = 139; *M*_age_ = 23.04; 50% women, 50% men). We conducted a serial mediation wherein COVID-19 racism predicted alcohol use severity sequentially through depressive symptoms and drinking to cope motives. COVID-19 racism directly and significantly predicted alcohol use severity. The indirect effect via depressive symptoms and drinking to cope motives was also significant, suggesting that COVID-19 racism is likely a risk factor for alcohol-related problems. Results inform intervention science and highlight the need for policy and behavioral health services to curb COVID-19 racism.

COVID-19 has reanimated and intensified anti-Asian racism in the U.S. The World Health Organization warned against increasing racist stereotypes and stigma against people of Asian ancestry vis-à-vis misinformation regarding the COVID-19 outbreak (Turner-Musa et al., 2020). A social media sentiment analysis found that anti-Asian racist tweets increased by 68% from November 2019 to March 2020 when the initial outbreak occurred (Nguyen et al., 2020). Polarization of these racist tweets on the internet (Keum & Miller, 2018) synonymized COVID-19 with Asian racial identity (e.g., “China virus,” “Kung-flu”) and pathologized people of Asian ancestry as vectors for precipitating and proliferating the pandemic (Chen et al., 2020). Time series analysis revealed that implicit bias against Asian Americans began increasing in March 2020 following the propagation of racially stigmatizing language by conservative media outlets (Darling-Hammond et al., 2020). Critical scholars have contextualized the racialization of Asian Americans—as biological, cultural, and societal pathogens and existential threats to white supremacy—as a state-sanctioned phenomenon that, throughout U.S. history, provided racist pretexts for significant human rights violations and other legal injustices against this population (Gover et al., 2020; Lee, 2021; Mallapragada, 2021). COVID-19 racism is a nontrivial behavioral health threat to Asian American emerging adults (aged 18–29) as it may engender and exacerbate racial impediments to an otherwise crucial developmental period (Daw et al., 2017)—for exploring and integrating personal identities, consolidating executive and self-regulatory competencies, and establishing lifespan trajectories in terms of interpersonal relationships, career decisions, and civic and community engagement.

COVID-19 racism has manifested in verbal, physical, and structural violence. In a national study (Dhanani & Franz, 2020), over 40% of U.S. adults admitted that they would perpetrate at least one discriminatory behavior toward people of Asian ancestry. According to the Stop AAPI Hate Reporting Center, Asian Americans have been verbally and behaviorally harassed, avoided, shunned, physically assaulted, coughed and spat on, and excluded from job and career opportunities due to COVID-19 racism (Yellow Horse et al., 2021). Multiple U.S. national studies have likewise documented increased anti-Asian racism and xenophobia in the general population (Gao & Liu, 2021), including reported hate crimes across major cities (Levin, 2021). By September 2021, the Stop AAPI Hate Reporting Center received over 10,000 reports of anti-Asian hate incidents across the U.S. (Yellow Horse et al., 2021). Concerningly, evidence indicates the continued and unabated perpetuation of COVID-19 racism across online settings (He et al., 2021), schools (Le et al., 2021), and workplaces (Gardner et al., 2021).

Experiencing COVID-19 racism is linked to multiple psychosocial health problems (Saw et al., 2021), including anxiety and depressive symptoms (Chae et al., 2021; Cheah et al., 2020), post-traumatic stress (Ashby et al., 2021; Hahm, Ha, et al., 2021), and psychological distress (Hahm, Xavier Hall, et al., 2021). COVID-19 racism is racially traumatic for many Asian Americans (Liu & Modir, 2020; Saw et al., 2021; Yang et al., 2021). Consistent with Carter’s (2007) race-based traumatic stress model—where systemic racism confers chronically traumatogenic behavioral health consequences—COVID-19 racism may exacerbate social avoidance, autonomic hyperarousal, intrusive symptoms, other negative affective and cognitive alterations, and the consequent risk of developing maladaptive coping behavior (e.g., substance use) to regulate emotional distress. Racial discrimination may also precipitate depressive symptoms by motivating internalized self-negativity, diminished self-worth, and disempowerment given one’s dehumanized and marginalized racial status in a white supremacist society (Brondolo et al., 2016). The literature thus suggests that for Asian Americans, COVID-19 racism represents an emergent social determinant of behavioral health, a contemporaneous form of structural stigma (Hatzenbuehler, 2016), and an ecological risk factor for negative health outcomes (Chen et al., 2020; Hahm, Ha, et al., 2021) and population-level racial health inequities (Saw et al., 2021, 2022; Wu et al., 2021).

In this study, we examined whether COVID-19 racism predicted alcohol use severity via depressive symptoms and drinking to cope motives among Asian American emerging adults—a crucial population for this line of inquiry. Meta-analyses show that racism is associated with increased psychiatric vulnerability among Asian Americans (Paradies et al., 2015) and emerging adults of color (Vines et al., 2017). Asian American emerging adults are already a higher risk population for alcohol-related problems (Grant et al., 2004, 2017; Hai et al., 2021), a concerning trend that COVID-19 racism may exacerbate (Saw et al., 2021), as coping with race-related stress is a known motivator for alcohol use among Asian Americans (Chae et al., 2008; Iwamoto et al., 2022). Finally, behavioral risk factors—including substance use—established in adolescence are known to prefigure adult health consequences in the general population (Centers for Disease Control and Prevention, 2016). Considering these findings jointly highlights the need for research that empirically addresses the psychiatric implications of the current sociopolitical context on Asian American emerging adults.

## COVID-19 Racism, Depressive Symptoms, and Alcohol Use Severity

COVID-19 racism may accelerate the already growing overall risk of alcohol-related problems among Asian Americans. Contravening popularized “model minority” stereotypes (Chou & Feagin, 2015) and the historical erasure of Asian Americans from racial health disparities research (Yi, 2020), problematic alcohol use among Asian American emerging adults is increasing (Grant et al., 2004; 2017). An analysis of the 2015–2018 National Survey on Drug Use and Health found that this population exhibited a high risk for alcohol misuse and alcohol use disorder, particularly among U.S.-born Koreans, Filipinos, and Indian Americans (Hai et al., 2021). Dependence, excessive use, and abuse of alcohol are associated with multiple long-term health consequences, including heart disease, immune deficiency, liver damage, and other cognitive, psychiatric, and social impairments (World Health Organization, 2019). Hence, better understanding the substance use psychopathology of COVID-19 racism is imperative.

Racial discrimination, depressive symptoms, and increased risk for alcohol abuse are linked among Asian Americans (Iwamoto et al., 2016). Here, depressive symptoms and drinking to cope motives may be important operative factors between COVID-19 racism and alcohol use severity (Iwamoto et al., 2022; Saw et al., 2021). For example, the tension-reduction model (Brondolo, Ver Halen, et al., 2009) suggests that racially minoritized people may use alcohol to mitigate race-related stress and related psychological disturbances, a pattern confirmed empirically across several studies (Grekin, 2012; Wei et al., 2010). Furthermore, corroborating work has found that using alcohol to cope with race-related stress is a risky behavior (Gilbert & Zemore, 2016) that can potentiate long-term racial health disparities (Paradies, 2006).

Drinking to cope motives are indeed salient among Asian Americans experiencing race-related stress (Chae et al., 2008; Iwamoto et al., 2022) with greater risk likely for those with established problematic alcohol use. Lee-Won et al. (2017) reported that the anger and shame following exposure to anti-Asian racist tweets precipitated drinking to cope motives among Asian Americans. Le and Iwamoto (2019) found that among Asian American college students, racial discrimination was associated longitudinally with alcohol-related problems, mediated by drinking to cope motives. Iwamoto et al. (2022) demonstrated sequential associations among everyday racism, psychological distress, drinking to cope motives, and alcohol-related problems among Asian American college students. COVID-19 racism and depressive symptoms thus appear to represent distal risk factors that predict drinking to cope motives—a proximal risk factor to actual alcohol use (Kuntsche & Kuntsche, 2009).

## Study Purpose

Our review suggests collectively that depressive symptoms associated with COVID-19 racism may increase drinking to cope motives among Asian American emerging adults. Emerging adulthood is a developmentally critical and sensitive period where numerous converging factors can contribute to alcohol use. For those without robust self-regulatory competencies and contextual supports, increased psychological stress from adjustment challenges or adverse life experiences can promote alcohol use and dependence (Daw et al., 2017). Coping-related drinking may be particularly salient for Asian American emerging adults given the normalization of detrimental alcohol use in some Asian ethnic cultures (Cook et al., 2012) as well as peer norms and social contexts that foster alcohol use, such as in college and university settings (Iwamoto et al., 2016). For instance, Kenney et al. (2018) found that postsecondary students with higher depressive symptoms and perceptions of heavy drinking among their peers reported increased alcohol consumption and related consequences. Drinking to cope motives are a known mediator between depressive symptoms and alcohol use outcomes among college students more generally (Bravo et al., 2018; Villarosa et al., 2018). Determining how COVID-19 racism, depressive symptoms, and alcohol use are related in this population is thus a crucial question for public health surveillance and intervention (Misra et al., 2020).

**Figure 1.**
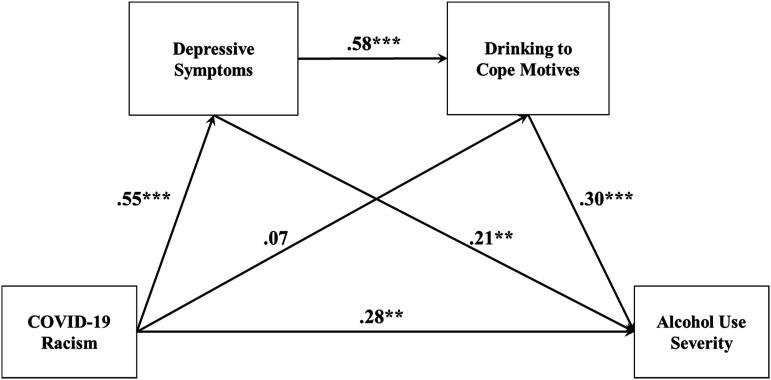
Estimated path model. *Note*. **p < .01 ***p < .001.

We tested whether COVID-19 racism predicted alcohol use severity via depressive symptoms and drinking to cope motives among Asian American emerging adults. We hypothesized that the frequency of COVID-19 racist incidents would predict greater alcohol use severity through greater depressive symptoms and drinking to cope motives, respectively. We path modeled (Figure 1) the following hypotheses:

Hypothesis 1 COVID-19 racism will directly predict alcohol use severity.

Hypothesis 2 COVID-19 racism will also indirectly predict alcohol use severity via depressive symptoms and drinking to cope motives (greater COVID-19 racism -> greater depressive symptoms -> greater drinking to cope motives -> greater alcohol use severity).

## Method

### Participants

We derived convenience data from 139 Asian American emerging adults (*M*_age_ = 23.04, *SD* = 2.13). About 28% (*n* = 39) identified as Chinese, 15% (*n* = 21) as Asian Indian, 12% (*n* = 16) as Filipino, 9% (*n* = 12) as Vietnamese, 8% (*n* = 11) as Japanese, 6% (*n* = 9) as Korean, 2% (*n* = 3) as Thai, 2% (*n* = 3) as Taiwanese, 1% (*n* = 2) as Bangladeshi, 1% (*n* = 2) as Indonesian, 1% (*n* = 2) as Hmong, 1% (*n* = 1) as Laotian, 1% (*n* = 1) as Singaporean, 1% (*n* = 1) as Cambodian, 9% (*n* = 12) as bi/multiethnic (between Asian ethnicities), and 3% (*n* = 4) as other. About half identified as women (*n* = 70) and men (*n* = 69), respectively. About 74% (*n* = 103) indicated being born in the U.S. and 26% (*n* = 36) reported being foreign-born. About 45% (*n* = 62) reported having a bachelor’s degree, 14% (*n* = 19) completing high school, 12% (*n* = 17) having an associate degree, 12% (*n* = 16) completing some college, 9% (*n* = 14) having a master’s degree, 4% (*n* = 5) having a doctoral degree, 2% (*n* = 3) completing a post-baccalaureate, 1% (*n* = 2) completing less than high school, and 1% (*n* = 1) other. About 58% (*n* = 81) reported having just enough money for their needs, 25% (*n* = 35) reported having not enough money to meet their needs, and 17% (*n* = 23) reported having more money than they need.

### Procedure

After acquiring Institutional Review Board approval, we recruited participants from June to July 2021 using Qualtrics Panel Service, which samples a targeted population of respondents nationwide from various sources, including website intercept recruitment, member referrals, targeted email lists, gaming sites, customer loyalty web portals, permission-based networks, social media, etc. We invited participants to informed consent and respond to an online survey about Internet experiences. The study inclusion criteria were: (1) being 18–29 years old, (2) self-identifying as Asian American, and (3) residing in the U.S. The survey comprised demographic and study variable measures and attention check items. Participants completed the survey within 15–20 minutes and received compensation up to $10 in the format (e.g., cash, gift cards, rewards points, etc.) concordant with the venue of their recruitment. 

### Measures

#### COVID-19 Racism

We adapted the COVID-19 racial discrimination items from Cheah et al. (2020) who modified four items from the Asian American Racism-Related Stress Inventory (Liang et al., 2004) to assess exposure to COVID-19-related racist messages and behaviors. Participants respond on a 6-point Likert scale (1 - *never* to 6 - *every day*). The items are: “Someone said something negative about Asian people (e.g., their diet) related to the COVID-19 outbreak,” “Someone said something about avoiding places with Asian people because of the COVID-19 outbreak,” “Someone tried to avoid me because I am Asian during the COVID-19 outbreak,” and “I felt self-conscious in public because I was worried about how others may think of me due to the COVID-19 outbreak.” Scores are summed with higher total scores indicating greater exposure to COVID-19 racism. Cheah et al. (2020) reported adequate internal consistency (α = .84–.86) and validity evidence (e.g., positive association with poorer mental health) of these items among Chinese American families.

#### Depressive Symptoms

We used the Patient Health Questionnaire-9 (PHQ-9) to assess the severity of depressive symptoms (Kroenke & Spitzer, 2002). Participants respond to nine items on a 4-point Likert scale (0 - *not at all* to 3 - *nearly every day*). Summed scores range from 0 to 27 where higher scores indicate more severe symptomatology. Keum et al. (2018) reported strong reliability (α = .86–.93), validity evidence (e.g., positive relation with alcohol use), and measurement invariance of the PHQ-9 with Asian American emerging adults.

#### Drinking to Cope Motives

We used the 3-item coping subscale from the 12-item Drinking Motives Questionnaire-Revised Short Form (DMQ-R SF; Kuntsche & Kuntsche, 2009) to assess drinking to cope motives in the past 12 months. Participants respond on a 5-point Likert scale (1- *never* to 5 – *always*). A sample item is: “…because it helps you when you feel depressed or nervous.” Scores are summed with higher total scores indicating greater motives to drink. The DMQ-R SF has evinced strong reliability (α = .92) with Asian Americans (Iwamoto et al., 2022) and positive relations with alcohol use and alcohol-related problems (Kuntsche & Kuntsche, 2009).

#### Alcohol Use Severity

We used the 10-item Alcohol Use Disorders Identification Test (AUDIT; Saunders et al., 1993) to assess risky or harmful alcohol consumption and alcohol dependence and abuse. The AUDIT items represent alcohol consumption (items 1–3), drinking behavior/dependence (items 4–7), and alcohol-related problems or consequences (items 8–10), and are summed. The first eight items are scored on a 5-point Likert scale ranging from 0 to 4, and the last two are scored on a 3-point Likert scale with values of 0, 2, and 4. The total scores range from 0 to 40, with higher scores indicating more severe alcohol problems. The AUDIT has demonstrated adequate reliability with an Asian sample (α = .85; Oei & Jardim, 2007) and ample validity evidence—including positive relations with other alcohol measures (e.g., MacAndrew alcoholism scale), alcoholism vulnerability, and somatic and affective costs of drinking (Bohn et al., 1995).

### Data Analysis

There were no missing data. To determine the necessary sample size and statistical power for the hypothesized path model, we conducted a Monte Carlo simulation (Schoemann et al., 2017). We drew effect size estimates for each of the proposed paths (ranging from .30 to .50) informed by relevant prior studies that have reported moderate to large effects (Iwamoto et al., 2022; Villarosa et al., 2018). Results from 10,000 replications indicated that *N* = 73 was sufficient to achieve power ≥ .80 at α = .05. Coverage was 95% and parameter and standard error bias was < .05. Results suggested altogether that our study was adequately powered.

We path modeled our hypothesized serial mediation (Figure 1) using M*plus* 8.7 (Muthén & Muthén, 1998–2017) with maximum likelihood estimation with robust standard errors. We specified COVID-19 racism as the predictor, depressive symptoms and drinking to cope motives as sequential mediators, and alcohol use severity as the dependent variable. We controlled for gender and nativity as men consume more alcohol and experience more alcohol-related problems (White, 2020) and because U.S.-born Asians report more alcohol-related problems than those foreign-born (Iwamoto et al., 2016), generally speaking.

We evaluated model fit using the Yuan-Bentler (YB) scaled *χ*^*2*^ test and several approximate fit indices (Hu & Bentler, 1999): (a) the root mean square error of approximation (RMSEA; close to < .08 for “acceptable” fit); (b) the comparative fit index (CFI; > .95 for “good” fit); and (c) the standardized root mean square residual (SRMR; close to < .08 for “acceptable” fit). To examine specific path coefficients and indirect (i.e., mediation) effects, we followed best practices (Hayes & Scharkow, 2013) and adopted the bootstrap method using 5,000 random samples. We used 99% confidence intervals (CI) to assess the statistical significance of the mediation effects, considering CIs that excluded 0 equivalent to *p* < .01. Finally, we tested an alternative model with depressive symptoms and drinking to cope motives as parallel mediators and compared its fit against the hypothesized serial model, using the Yuan-Bentler (YB) scaled *χ*^*2*^ difference test and the Akaike and Bayesian information criterion (AIC; BIC) values.

## Results

Bivariate correlations, reliability estimates, and other descriptive statistics are in Table 1. We did not detect any evidence of violation to the univariate normality assumption (George & Mallery, 2010). Internal consistency estimates were high for all scales.

**Table 1.** Study Variables: Descriptive Statistics and Bivariate Correlations.

	Descriptives							Correlations		
Variables	Min	Max	*M*	*SD*	Skewness	Kurtosis	α	1	2	3
1. COVID-19 racism	4.00	24.00	10.19	5.30	.62	−.51	.88	-		
2. PHQ-9	0	27.00	10.51	6.68	.17	−.57	.90	.55^**^		
3. DMQ-R SF-C	3.00	15.00	7.93	3.72	.31	−1.03	.88	.39^**^	.62^**^	
4. AUDIT	0	32.00	11.01	9.21	.91	−.54	.90	.52^**^	.54^**^	.56^**^

*Note*. PHQ-9 = Patient Health Questionnaire-9 (Depressive Symptoms); DMQ-R SF-C = Drinking Motives-Revised Short Form Coping (Drinking to Cope Motives); AUDIT = Alcohol Use Disorders Identification Test (Alcohol Use Severity).

**p < .01.

### Serial Mediation Model

Our hypothesized serial model fit the data well, $\chi_{\text{YB}}^{2}\;$ = 6.735, *df* = 6, *p* = .35; RMSEA = .030 [.000, .117]; CFI = .99; SRMR = .027. The alternative parallel model fitted poorly, $\chi_{\text{YB}}^{2}\;$ = 47.622, *df* = 7, *p* < .001; RMSEA = .204 [.152, .261]; CFI = .77; SRMR = .078. The $\chi_{\text{YB}}^{2}\;$ difference test was significant and favored the hypothesized serial model, Δ$\Delta \chi_{\text{YB}}^{2}\;$ = 33.823, Δ*df* = 1, *p* < .001. The AIC and BIC also supported the serial model as its values (2510.66; 2507.22) were less by more than 10 units (Burnham & Anderson, 2004) compared to the parallel model (2554.46; 2551.25). Thus, we retained our hypothesized serial model and ruled out the alternative parallel model. Figure 1 illustrates a completely standardized path diagram. Table 2 reports the total direct, total indirect, and specific indirect effects.

**Table 2.** Serial Mediation Model: Bootstrapped Estimates of Model Effects.

IV	Mediator(s)	DV	Standardized Effect Estimate	*SE*	99% Bootstrapped CI
Total effect					
COVID-19 racism		AUDIT	.514**	.09	[.314, .713]
Total direct effect					
COVID-19 racism		AUDIT	.283**	.09	[.047, .518]
Total indirect effect					
COVID-19 racism		AUDIT	.231**	.05	[.111, .351]
Specific indirect effect					
COVID-19 racism → PHQ-9 →		DMQ-R SF-C	.318**	.06	[.174, .463]
PHQ-9 → DMQ-R SF-C →		AUDIT	.176**	.06	[.042, .310]
COVID-19 racism → PHQ-9 → DMQ-R SF-C →		AUDIT	.096**	.03	[.023, .169]

*Note*. IV = independent variable; DV = dependent variable; CI = confidence interval; PHQ-9 = Patient Health Questionnaire-9 (Depressive Symptoms); DMQ-R SF-C = Drinking Motives-Revised Short Form Coping (Drinking to Cope Motives); AUDIT = Alcohol Use Disorders Identification Test (Alcohol Use Severity).

**p < .01.

Overall, COVID-19 racism significantly predicted alcohol use severity (standardized effect *β* = .514, 99% bootstrapped CI = [.314, .713]). The total effect was decomposed into a significant direct effect (*β* = .283, 99% bootstrapped CI = [.047, .518]) and a significant total indirect effect through the hypothesized mediators (standardized total indirect effect *β* = .231, 99% bootstrapped CI = [.111, .351]) that explained 45% of the total effect. The indirect pathway from COVID-19 racism to drinking to cope motives via depressive symptoms was significant (standardized total indirect effect *β* = .318, 99% bootstrapped CI = [.174, .463]). The indirect pathway from depressive symptoms to alcohol use severity via drinking to cope motives was significant (standardized total indirect effect *β* = .176, 99% bootstrapped CI = [.042, .310]). The indirect pathway from COVID-19 racism to alcohol use severity via depressive symptoms and drinking to cope motives was significant (standardized total indirect effect *β* = .096, 99% bootstrapped CI = [.023, .169]). Thus, COVID-19 racism indirectly predicted alcohol use severity through depressive symptoms and drinking to cope motives sequentially. The model accounted for 48% of the variance in alcohol use severity.

## Discussion

COVID-19 racism is an ongoing public health emergency for Asian Americans. A growing literature illustrates the pervasive psychiatric implications of anti-Asian hate—manifested through verbal, physical, and structural violence. To extend this evidence base, we sought to confirm the associations among COVID-19 racism and two major and comorbid public health problems—depressive symptoms and risky alcohol use (Kessler et al., 2003; Room et al., 2005)—which represent unmet population health needs among Asian Americans (Iwamoto et al., 2016; Kim et al., 2015) and heretofore unexamined relations of COVID-19 racism. Our study is among the first to examine the serially mediating function of depressive symptoms and drinking to cope motives between COVID-19 racism specifically and alcohol use severity among Asian American emerging adults, an understudied and contemporaneously vulnerable population.

The results supported our theory-based hypotheses and were consistent with relevant findings from recent studies (Iwamoto et al., 2022). First, COVID-19 racism directly predicted alcohol use severity, denoting its immediate psychological impact. Moreover, COVID-19 racism predicted alcohol use severity indirectly through the sequence of first promoting depressive symptoms, which increased drinking to cope motives, and finally alcohol use severity. These patterns were consistent across gender and nativity, suggesting that the psychiatric cascade of COVID-19 racism holds some within-group uniformity in this population notwithstanding established subgroup differences in alcohol consumption and problematic use (Iwamoto et al., 2016). Notably, our findings also conformed with a prior notation regarding the depressant function of anticipatory race-related vigilance (Chae et al., 2021; Liu & Lau, 2013), as our measures included an item describing preemptive social anxiety about COVID-19 racism. The statistical and clinical significance of our analysis suggests that increased alcohol use severity among Asian American emerging adults may represent coping attempts against depressive symptoms precipitated by COVID-19 racism. This is alarming given possible neurodevelopmental disruptions to executive function and self-regulation otherwise coalesced during emerging adulthood, and the potential propagation of chronic alcohol use disorder and dependence and their long-term sequelae (Daw et al., 2017; Nixon, 2013).

Our results delineated an important psychological mechanism by which COVID-19 racism—arguably an evolving form of both race-based traumatic stress (Carter, 2007) and structural stigma (Hatzenbuehler, 2016) impacting Asian Americans—engenders affective disturbance and maladaptive coping responses in turn. Concerningly, the uninterrupted expansion of COVID-19 racism may portend a societal shift toward a “new normal” of anti-Asian hate and imply nontrivial psychiatric consequences for this population. Scholarship has noted how racialized Islamophobic violence post-9/11 has persisted and increased over time (Al Atom, 2014; Kishi, 2017) and conferred intergenerational and multilevel mental health impact on South Asian and SWANA populations (Samari et al., 2018; Sirin et al., 2021). Emerging national data have shown similarly that COVID-19 racism accounts partially for worsening mental health disparities between Asian and white Americans (Wu et al., 2021). Considering these historical precedents, deepening economic inequities, and global sociopolitical polarization (Elias et al., 2021), COVID-19 racism may presage an era of intensified yet normalized anti-Asian hate, institutional discrimination, and extralegal violence against Asian Americans. Implications for Asian American emerging adults include an amplified convergence (Iwamoto et al., 2022; Lee-Won et al., 2017) of developmental vulnerabilities in race-related stress (Paradies et al., 2015; Vines et al., 2017) and problematic alcohol use (Grant et al., 2004, 2017; Hai et al., 2021) that may potentiate long-term syndemic health disparities (Saw et al., 2022).

We urge policymakers to strengthen public health research infrastructures for reporting, monitoring, and predicting the trajectory of anti-Asian racism and its link to key behavioral health outcomes over the foreseeable future (Asian American Psychological Association, 2021). Asian Americans are among the most underrepresented populations in both funded (Ðoàn et al., 2019) and peer-reviewed (Ghosh, 2003) science, including alcohol use (Iwamoto et al., 2016) and health disparities (Yi, 2020) research. We call for increased institutional support for scholarship that centralizes contextual and cultural variables relevant to health care access and utilization, disparities, and promotion in this group (Le et al., 2020). Finally, advancing psychosocial interventions and services to address anti-Asian racial trauma and its intergenerational, psychiatric, and socioeconomic sequelae is crucial (Asian American Psychological Association, 2021; Liu & Modir, 2020). As a chronically underserved group in behavioral health services (Ihara et al., 2014; Sue et al., 2012)—including substance abuse treatment (Sakai et al., 2005)—and because perceived discrimination deters help-seeking (Burgess et al., 2008), Asian American emerging adults are an exceptionally vulnerable population at the present time, indicating proactive clinical practice efforts.

### Limitations and Future Research Directions

Our findings should be considered with several limitations. We used a convenience sample of Asian American emerging adults and a simple path model to substantiate the growing evidence base concerning the deleterious behavioral health function of COVID-19 racism. Our cross-sectional data precluded any assertion of temporal precedence and true time-sequential directionality. Given sample size limitations, we could not disaggregate our findings across ethnicity, gender, geographic region, and socioeconomic status represented diversely among our participants. As aforementioned, certain Asian ethnic groups are at a greater risk of alcohol-related problems, such as Korean, Filipinx, and Indian Americans (Hai et al., 2021). Available data show that women and older adults are overrepresented in reported anti-Asian hate incidents (Yellow Horse et al., 2021). Racial climate across geographic region may also moderate the severity of COVID-19 racism. Future studies should enlist longitudinal data with larger samples to examine ethnic, gender, and geographic differences and replicate our hypothesized directional relations among COVID-19 racism, depressive (and other psychiatric) symptoms, drinking to cope motives, and alcohol use severity. Future studies might extend our model by examining preexisting alcohol consumption patterns and intrapersonal factors (e.g., internalized racism, racial trauma symptoms) that may moderate depressive symptomatology and drinking to cope motives vis-à-vis COVID-19 racism. Similarly, we encourage work that analyzes other empirically confirmed social-cognitive correlates of alcohol use severity—such as alcohol values and expectancies and peer influence (Iwamoto et al., 2010)—as well as substance use behavior of known public health import for Asian American subgroups, such as tobacco (Lew & Tanjasiri, 2003; Rao et al., 2021). Finally, our framework should be expanded to test key cultural and intersectional variables (Keum et al., 2022; Keum & Choi, 2021)) germane to the link among COVID-19 racism and alcohol use outcomes, given that acculturative dynamics and gender appear to typify “high risk” drinkers among Asian American emerging adults (Cook et al., 2015; Iwamoto et al., 2019).

## Conclusion

COVID-19 racism is a pressing public health crisis and Asian American emerging adults are particularly vulnerable regarding race-based behavioral health outcomes. We advanced a framework by which COVID-19 racism may precipitate a psychiatric cascade of depressive symptoms, drinking to cope motives, and alcohol use severity in this population. Our serial mediation analysis showcased the multiple yet unambiguously harmful psychological pathways by which COVID-19 racism promotes affective distress and maladaptive coping behavior, but which may hold promise for clinical intervention (MacKinnon et al., 2007). Our findings contributed to the evolving research consensus regarding the nefarious association among COVID-19 racism and numerous population behavioral health problems affecting Asian Americans.
